# “Girls get stressed due to the situation we are facing”: Multi-level mental health stressors and resilience enablers among adolescent girls and young women in South Africa

**DOI:** 10.1371/journal.pmen.0000286

**Published:** 2025-05-28

**Authors:** Zoe Duby, Brittany Bunce, Kate Bergh, Nokuthula Cwele, Colleen Wagner, Caroline Kuo, Fareed Abdullah

**Affiliations:** 1 Health Systems Research Unit, South African Medical Research Council, Cape Town, South Africa; 2 School of Public Health, University of Cape Town, Cape Town, South Africa; 3 Universitat Autònoma de Barcelona, Barcelona, Spain; 4 Department of Psychology, University of Cape Town, Cape Town, South Africa; 5 Innovation Guru, Pretoria, South Africa; 6 Networking HIV/AIDS Community of Southern Africa (NACOSA), Cape Town, South Africa; 7 American University, Washington, DC, Unites States of America; 8 Department of Psychiatry and Mental Health, University of Cape Town, Cape Town, South Africa; 9 Office of AIDS and TB Research, South African Medical Research Council, Pretoria, South Africa; 10 Faculty of Health Sciences, University of Pretoria, Pretoria, South Africa; Sigmund Freud University Vienna, AUSTRIA

## Abstract

Poor mental health, including high levels of stress, is associated with increased risk behaviours and subsequent negative sexual and reproductive health (SRH) outcomes amongst adolescent girls and young women (AGYW). Multilevel mental health stressors reflect various levels of AGYW’s social ecology. We use the social-ecological model as a framework to analyse qualitative data collected through interviews with 51 AGYW in two South African communities characterised by high rates of HIV, early pregnancy and school drop-out. We explore AGYW’s narratives of stressors, coping strategies, and impacts of participating in an intervention on mental well-being. Individual level stressors included low self-esteem, non-heteronormative sexuality, academic stress, and early child-bearing. Interpersonal level stressors included perceived lack of psychosocial support, emotional isolation, communication barriers, peer pressure and bullying. Microlevel stressors at the family or household level included violence, conflict and substance use. Structural level stressors included household food insecurity, economic hardship and gendered inequalities. Coping strategies and resilience enablers at the individual level included maintaining a positive outlook, engaging in self-care activities and spending time with family. Resilience strategies at the interpersonal level included seeking psychosocial and emotional support and talking about feelings, whilst resilience enablers at the macrosystem level included faith communities and access to social workers. Respondents who had participated in the intervention reported improved mental health and well-being due to increased access to psychosocial support. Intersecting factors across individual, interpersonal and structural levels are salient mental health stressors experienced by AGYW. However, some AGYW manage to draw on internal individual resources and external interpersonal and contextual resources to increase resilience and promote coping. Strengthening and bolstering resilience enabling resources available to AGYW, through enhancing AGYW’s competence and self-efficacy in accessing psychosocial support, alongside ensuring that support is available, may be important components to include in future interventions to support AGYW mental wellbeing.

## Introduction

There is strong evidence of how poor mental health, inclusive of depression, anxiety, trauma and stress, increases risk behaviours amongst adolescents and young people. Sexual risk behaviours can increase when mental health is poor, and in turn, are associated with negative sexual and reproductive health (SRH) outcomes such as HIV infection, inconsistent use of contraceptives, early and unintended pregnancy, and unsafe abortion amongst adolescent girls and young women [1–3]. An individual’s mental health status during the period of adolescence affects their education, future health, wellbeing and opportunities [1,2,4]. Multiple situational, socio-economic, environmental and contextual factors impact the mental well-being of adolescents and young people [1]. Adolescents and young people who grow up in socio-economically restricted settings face various mental health stressors including higher levels of food insecurity, violence, substance use, and limited psychosocial support [1,2,5].

Psychological distress has been defined as a state of emotional suffering and discomfort, a subjective negative feeling perceived by individuals in response to events or circumstances – ‘stressors’ that they feel exceed their resources to cope [6–9]. Psychological distress is often a precursor to anxiety, depression, and suicidal ideation, and is associated with a range of non-communicable diseases such as cardiovascular and respiratory complications, malnutrition and poor SRH outcomes [2,6,7,10].

### Theoretical framework

Adaptations of the social-ecological model, Bronfenbrenner’s Ecological Systems Theory of Human Development (1979), which examines interactions and relationships between individuals and their social and environmental context, have been applied to examine the multilevel influences on adolescent mental health and well-being. The model places the adolescent at the centre of circles of influence: individual/ intrapersonal factors such as age, gender, and personality characteristics; then interpersonal factors, such as family and home environment, school and peers, at the microsystem level; expanding out to socio-cultural values and norms, as well as health and education policies and institutions, at the exosystem and macrosystem levels [11–14]. Employing a socio-ecological perspective, an individual adolescent’s mental health can be understood as the product of multiple interacting and intersecting levels of influence [15]. Using this model to examine multilevel mental health stressors can help to ensure that mental health interventions for adolescents are responsive and relevant to contextual needs [11].

Lazarus and Folkman’s (1984) theory of Stress and Coping helps to explain how individuals interpret subjective perceptions of psychological distress, alongside perceptions of coping. This theory frames psychological distress as a dynamic process, and the way in which an individual experiences psychological distress is dependent on their own interpretation and appraisals of stressors, responses to, and capacity to cope with them [2,16]. In contrast to the negative response to stressors that some individuals have, there has been growing interest in why other individuals, faced with the same stressors and adversity, manage to cope, and even thrive, despite the adversity – this is referred to as ‘resilience’ [17]. Resilience should not be understood as an individual’s invulnerability to stress, but rather an ability to cope with stressors [18]. An individual’s ability to cope with and respond to their feelings of psychological distress is crucial in mitigating the negative effect of stressors on their mental health and well-being [8].

### Rationale for this study

Evidence suggests that levels of psychological distress amongst adolescents have been steadily increasing, particularly amongst adolescent girls [19]. Given this context, there has been a growing acknowledgement of the need to integrate mental health components into broader SRH programming for adolescents and young people in South Africa [1]. In order to ensure that such interventions are appropriate and relevant there is a need to understand the perceptions of psychological distress as well as the factors that generate psychological distress amongst the intended intervention beneficiary populations themselves [2,7]. By building an understanding of the sources and factors to which young people ascribe their psychological distress to preventive interventions can be strategically tailored to meet their specific needs and thereby curb the psychological distress that adolescents experience in their daily lives, which in turn can help to reduce related outcomes such as school drop-out and sexual risk behaviours [4,19]. Whilst there have been studies examining the social, economic, environmental, physiological and interpersonal factors that contribute to negative mental health outcomes amongst young people in South Africa, there has been scant focus on the narratives of young people themselves on the multilevel factors that impact on their mental health and well-being.

In this paper we present analyses of qualitative data collected through interviews conducted with AGYW in two South African communities characterised by high rates of HIV, early and unintended pregnancy and school drop-out. Interviews were conducted prior to, and in the first months of a combination sexual and reproductive health intervention that included mental health components, being implemented in schools in these communities. Analyses presented focuses on adolescent girls and young women’s narratives and subjective experiences of psychological distress and stressors, strategies for coping, and impacts of participating in the intervention on mental well-being. We use the term ‘psychological distress’ to describe broadly the terms of anxiety, depression, trauma, and stress.

### Background to the intervention

**Fig 1:**
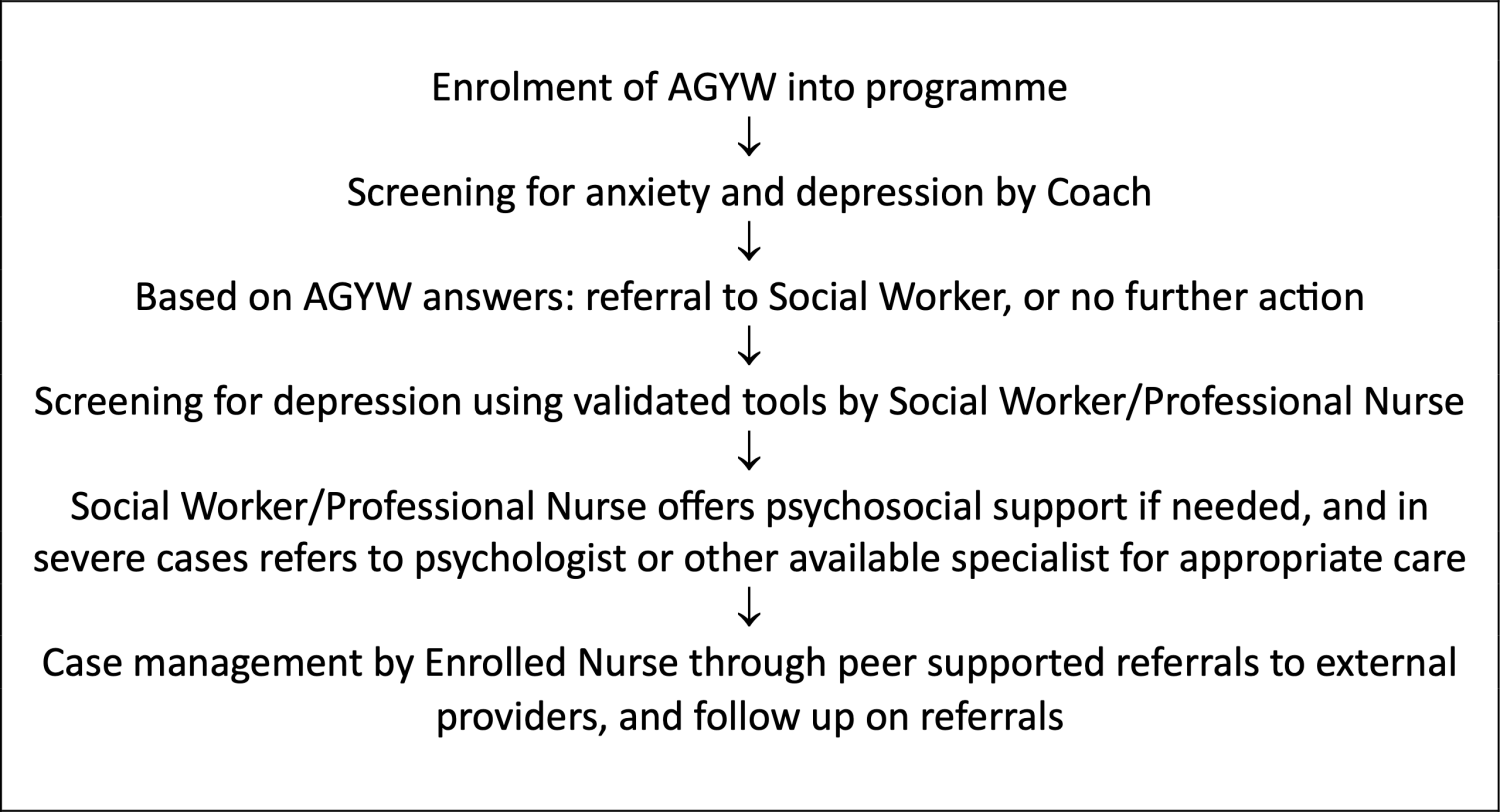
Imagine Programme Mental Health Screening Procedure.

The initial design of the Imagine Programme [20] included mental health components (see Fig 1) such as mental state screening and screening for depression, suicide and grief and with individual psycho-social counselling and/or referral for specialized services as needed. Coaching provided as part of the intervention included tools such as discussion cards and games designed to build rapport and assess AGYW needs and discuss emotions, mental health and goal setting. Additional psycho-social support was provided for those AGYW identified in need by programme Social Workers, or referrals to affiliated service providers in the community were made. At the time of data collection for this study, the Imagine Programme Safe Spaces provided AGYW with access to psychological services and were staffed by trained social workers and coaches who conducted assessments to determine if AGYW require individual or group counselling.

A baseline survey [21] conducted prior to the Imagine programme in the communities in which the intervention was to be implemented used the 10-item Centre for Epidemiological Studies Depression Scale (CES-D-10) to measure participants’ risk of depression. The findings showed that 39.1% of the total of 2353 survey participants were at a high risk of depression suggesting a need for mental health interventions [22].

## Methods

### Ethics statement

All AGYW who took part in interviews provided informed consent for participation. For those under 18 years of age, consent from a parent or legal guardian was obtained first. Consent procedures were conducted in participants’ preferred language. Interviewers read out the consent information to participants, who were provided the opportunity in each section to ask questions. Interviewers underwent ethics training, to ensure voluntary consent was ethically obtained. Participants provided oral consent, which was audio recorded, and saved on secure servers labelled with participant identification numbers. Participants received R150 (US $8) reimbursement for each interview they participated in. Ethical approval to conduct this study was obtained from the Human Research Ethics Committees at the South African Medical Research Council, and the University of Cape Town (EC045–10/2021).

As shown in Table 1, total of 51 AGYW participated in individual in-depth interviews (IDIs) over the period 01 July 2022–20 December 2023. In the first phase of data collection, which occurred before implementation of the Imagine Programme had commenced during the preparatory phase of the programme, 35 AGYW were interviewed between 01 July 2022 and 26 May 2023. A further 16 AGYW (not the same participants) were interviewed in the second phase shortly after the Imagine Programme implementation activities had been initiated, during the period 16 August to 20 December 2023. To be eligible to take part in interviews, AGYW had to be in grades 8–12 at sampled schools, able to speak one of the study languages, and willing and able to provide consent. Those AGYW younger than 18 years had to provide contact details of a parent/caregiver who could provide consent for the AGYW to participate, which was obtained telephonically and audio recorded. If a parent/caregiver did not grant consent, AGYW were not able to participate.

**Table 1:** Study Sample.

	Pre-intervention formative		Implementation phase	
	Moretele, North West	Newcastle, KwaZulu-Natal	Moretele, North West	Newcastle, KwaZulu-Natal
**Sample Group**				
AGYW 15–19 years	10	17	6	9
AGYW 20–24 years	4	4	0	1
TOTALS	14	21	6	10
***Total AGYW interviewed: 51***	**Total pre-intervention: 35**		**Total intervention phase: 16**	

Respondents were recruited from schools which had been selected for Imagine Programme implementation. Further details on the study sites and selection of schools has previously been reported [21]. As outlined in Table 2, study staff visited schools, and through liaising with school staff and Imagine Programme staff, made contact with AGYW and invited them to participate in interviews. Interviews were conducted by two female interviewers fluent in the local languages: Setswana and Sesotho in Moretele, and isiZulu in Newcastle, who were familiar with the local areas and cultural contexts, and were situated in the study communities for the duration of data collection. Interviews followed semi-structured topic guides and included the following questions relating to mental health: 1) In your words, how would you describe the things in your life that bring you the most happiness?; 2) What things in your life are you worried about?; 3) How would you describe the things in your life that bring you the most stress?; 4) How do you manage the stress that these things cause you?; and 5) How could this stress be reduced? Implementation phase participants were also asked: Could you tell us about any counselling you received as part of the programme? Interviews were conducted in participants’ language of choice and audio-recorded with participants’ consent. Interviews were conducted in safe, private and confidential spaces at schools or in the community. Audio recordings of interviews were transcribed and translated into English.

**Table 2:** Study Activity Flow.

Time period	Study activities		
June 2022 ↓	Relationship building at sampled schools, introducing study, recruitment		
July 2022 – May 2023 ↓	**Pre-intervention formative phase:** Eligible AGYW recruited and interviewed N = 35	Data processing	Preliminary data analysis
June 2023 ↓	Implementation of Imagine Programme starts in schools	Data processing	On-going data analysis
August – December 2023 ↓	**Implementation phase:** Eligible AGYW recruited and interviewed N = 16	Data processing	Data analysis & write up
January – February 2024	End of data collection	Data processing	Data analysis & write up

Transcripts were analysed using a collaborative iterative analytical process in which four data analysts collaboratively organized and coded data, working on evolving analytical memos, capturing emergent themes. Thematic analysis included a combination of inductive and deductive processes. Initially, predetermined deductive code types based on the topics included in the interview guides were used as a preliminary framework. Using a cyclic and iterative process, codes and themes were built upon through inductive development and refinement. Collaborative analysis of qualitative data enabled the integration of analysts’ diverse perspectives, which in combination serves to enhance the rigour of data interpretation [23–25]. Continuous dialogue and memo-ing was used in order to allow analysts to reflect on generative themes and interpretations, and attain agreement and consensus between analysts [24].

### Findings

**Fig 2:**
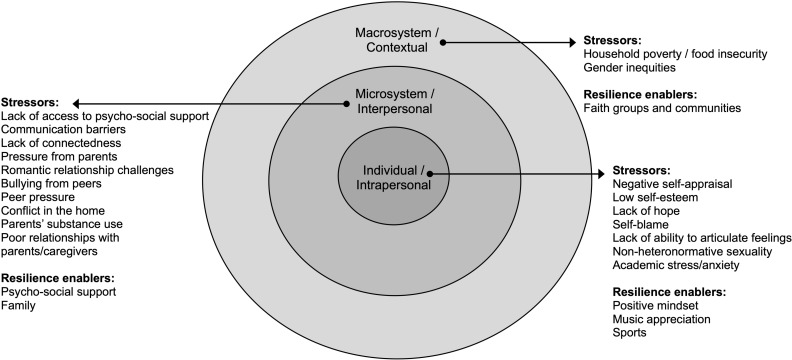
Individual/ Intrapersonal level stressors.

Fig 2 presents the mental health stressors and resilience enabling resources described by respondents in our study organised according to socio-ecological levels.

In participants’ narratives, negative self-worth, including low self-esteem, self-judgment and self-blame were described as harmful to AGYW mental health and well-being: “Our problem is that we focus too much on the mistakes we have done. We blame and judge ourselves even before other people can blame us” (Newcastle, 18years+). Low self-esteem and lack of self-belief contributed to lack of hope. This was especially the case for AGYW who get pregnant or bear children and lose hope for their future prospects. It was suggested that this loss of hope leads to maladaptive coping to psychological distress such as problematic alcohol use: “We as young women, have low self-esteem, if a young girl has a child, it will be like the world has come to an end and there is nothing she can do… She will drop out of school and she will not even look for any job opportunities. We have lost hope for the future as girls… We end up drinking alcohol because it helps us to release the stress that we are having” (Newcastle, 18years+).

Unexpected and unplanned pregnancies and early childbearing can cause anxiety and stress, and negatively impact on education, and as a result, AGYW struggle to cope with life’s challenges and pressures: “Teenage pregnancy… school and a child they do not go hand in hand… you will find yourself having problems” (Moretele, 18years+). In addition, a self-perceived lack of skills and capacity to be a parent creates compounding stress for AGYW: “They also have challenges with their kids… because they had these children at a young age” (Newcastle, 18years+).

It was suggested that many AGYW lack knowledge and information about mental health and where to access support, and they also lack the skills and language to talk about their feelings and needs. As a result, AGYW feel emotionally isolated and unsupported, which, in some cases, can result in depression and suicidality: “Girls of nowadays, when a person has a problem (and) they do not talk - you know for her the solution is to kill herself… it is not a solution to kill yourself when you have a problem… you know in life there will be someone who you trust, so like at least when you have a problem try to talk to someone and explain your problems to them… what kind of situation you’re in, and what can you do with your problems” (Moretele, 18years+). Respondents explained that they do not know who to confide in which exacerbates their emotional isolation: “Maybe if you have a problem, you don’t speak up, so you do not get the support… okay I might need someone to talk to but then… I don’t feel comfortable… to talk” (Moretele, 13–17 years).

It was suggested that sexuality can also be a cause of stress amongst AGYW. Those AGYW who do not fit into the heteronormative expectations of the community, particularly in contexts of homophobia, experience a lack of acceptance of their sexuality: “Some girls are… afraid to come out of the closet just because the community okay knows there’s a man and a women fact… so if you’re bi (bisexual), gay… homo (homosexual)… that’s really bad” (Moretele, 13–17 years). In some cases, families and parents are not accepting or understanding of the sexuality of AGYW, as narrated by one respondent: “I had a challenge at home… as you can see me I am a lesbian… So at home they are homophobic and that affected me because at home they are not accepting my situation” (Newcastle, 13–17 years).

Stress related to school and educational achievement was also a key concern cited by respondents. Many AGYW suggested that they feel stressed when struggling with homework or comprehending course content. Students feel weighed down by academic pressure and experience stress due to fear of failure at school and in exams. Respondents suggested that they get stressed by the volume of homework they have, with some saying that they struggle to balance having enough time for household chores with the time needed for homework. The self-perceived inability to cope with the academic workload was described by respondents as one of the sources of mental distress and anxiety: “Work load… from school gives you stress… they give us loads of work, they give us pressure… You get stressed… things get mixed up… they don’t work out… Mentally I would say school things, there’s a lot of pressure” (Moretele, 13–17 years).

### Microsystem/ Interpersonal level factors

Interpersonal level factors and relationship dynamics were another theme in respondents’ narratives of mental health stressors. The lack of access to sources of psycho-social support was a key aspect, with AGYW describing the feeling of lacking trusted people to confide in, creating a sense of being emotionally isolated. Narratives highlighted the lack of trusted support mechanisms and specialised trained counselling. Communication barriers combined with a lack of connectedness to caring and supportive adults such as teachers and parents further prevent AGYW accessing support: “It is not easy (for girls to get support they need)... They are afraid to talk to parents and teachers. We are afraid to talk to each other as girls… They end up being hurt for suppressing all those feelings” (Newcastle, 13–17 years).

One factor compounding AGYW feelings of lacking psychosocial support is poor relationships with parents/ caregivers. Communication gaps between AGYW and their parents mean that AGYW feel that they are not getting the support they need from their homes and caregivers. In some respondents’ narratives, the absence of parental guidance was identified as a significant source of stress as AGYW lack capacity to solve problems on their own. AGYW often feel unable to handle life’s stresses and feel that parents don’t give space to AGYW to talk about their problems. In addition, some AGYW feel that parents pressure them without giving guidance: “Many of us, we are pressured by parents… There are some parents who do not care for (what) their children… want to do… ‘Why are you doing that? It doesn’t suit you, do something like this and that…’… or (the child says) I want to be a doctor ‘Why do you want to be a doctor, be this and that’… that is when you get stressed to a point where you want to kill yourself, many young people kill themselves because of the pressure from parents… many of us are pressured by parents… they pressure us a lot… so I cannot say we get support… we do not get it… parents should provide guidance… if parents can communicate with children to say…‘the world it’s like this… you have to do so and so’… that is where we can succeed in life” (Moretele, 18years+).

Lacking psychosocial support during the difficult time of adolescence leaves AGYW more vulnerable to depression and unable to cope with life events and relationship stresses. For example, the end of romantic relationships can sometimes lead to mental distress for AGYW, to the extent where some become suicidal: “Some girls are just depressed over guys… after a break up… ‘Oh my God I love him why?’ [Laughing]… it is (enough) for her to kill herself... depression and all those (things)” (Moretele, 13–17 years).

One major interpersonal realm that was described as a stressor by respondents related to peers. Several aspects were cited, including conflictual relationships with peers in the school environment and bullying, as well as receiving negative and body-shaming comments from boys. Fear of gossip in the community, school and neighbourhood also creates stress for AGYW: “My neighbours always gossip about me since last year… They say I get pregnant and then do abortions… I normally cry and stay in my room alone… They said all these things last year, and by that time I was still a virgin… I had a problem, my stomach used to get bigger sometimes and becomes normal in other times, so seeing my tummy big they said I was pregnant and when it came back to its shape, they said I have done abortions” (Newcastle, 13–17 years).

The pressure of trying to fit in was described as a key mental health stressor by AGYW, who explained the lengths they go to avoid being judged by others, inclusive of needing to dress a certain way: “You’re trying to be the perfect person… it can cause a lot of stress… how people look at you, like how people are judgmental… you don’t want people to judge you (Moretele, 13-17 years). Being regarded as fashionable and fitting in to the peer group was of great importance to AGYW. This often led to wanting clothes and material goods that parents are unable to afford. At times these desires lead AGYW to engage in risk behaviours in an effort to impress peers: “We always have something that is occupying our minds… We always want better and expensive things, we think of what we will be wearing… where will we get the money?… (A cause of stress is that) we want to fit and we want to do things that are in fashion and we want things that parents cannot afford to buy for us… Maybe friends are planning a trip and if you do not have money, you will then have to think on where to get money… a girl might end up doing everything they say because she wants to impress her friends” (Newcastle, 18years+). Intersecting with macrolevel economic factors, peer pressure was tied up with attainment of material goods in the context of poverty, and AGYW explained that they want expensive items and will go to great lengths to ensure access to them, which can impact negatively on mental health: “We have stress at the younger age because we want fancy things and we end up getting into troubles because of that” (Newcastle, AGYW 13–17 years). Linked to the theme of materialism and the desire to fit in, AGYW experience stress due to material aspirations that cannot be met. This leads to risk behaviours including engaging in transactional sex to meet material needs/wants.

Conflict between family members and/or parents also has a negative impact on the mental health of AGYW. When there are situations of domestic violence or fighting in the home, AGYW witnessing such conflict experience anxiety, depression, and stress. Parents’ own sexual and romantic relationships and partners can also create difficult home situations, leaving AGYW feeling stuck and helpless. In situations where parents/caregivers engage in problematic alcohol consumption or have alcohol use disorders, AGYW mental well-being is negatively affected: “Things which stress me in my life… my mother the way she is drinking… it affects me too much because I cannot concentrate, I have to cook for the children, I have to clean, she wants everything to be perfect… I’m going to be strong, and I already told myself that I’m strong and there’s nothing I could fail to do, I will not fail to do it as long as God is with me and watching over me… I will win [sigh]” (Moretele, 13–17 years). Experiences of abuse or mistreatment at the hands of family members, step-parents or others in the household, compounded by a lack of support, further enhance AGYW sense of being isolated, causing some to become suicidal.

### Macrosystem level

In terms of contextual and structural level mental health stressors, AGYW explained that circumstances of poverty, food insecurity, poor health, and neglect all impact negatively on mental health. Family financial situations were described as a key stressor. In contexts such as these unpaid household labour including caring for younger children and elders often falls to AGYW, inhibiting their educational opportunities and their mental well-being: “Some girls get stressed due to the situation they are facing at their homes which might prevent them from going to school, maybe there is no food, no money at home… Others might tell you that they are staying at home to look after their sick parent, or they have children and there is no one to look after the children” (Newcastle, 18years+). Conflict with parents arose as a result of these gendered expectations and the burden of care that AGYW experience, particularly when AGYW struggle to attain a balance between homework and household duties: “There is a lot of pressure, you have to do your school work and do the house chores… At home they will tell you to do something whilst busy doing your school work and when you fail it becomes a problem too… It is difficult to have a balance… There is a lot of home work even if you have planned to study” (Newcastle, 13–17 years). Some AGYW articulated their views on the gendered inequalities favouring male siblings and family members, citing the pressure placed on AGYW to consistently contribute to household labour. These narratives also illustrate intergenerational struggles over the distribution of household labour, where cultural, gender and generational expectations intersect leaving AGYW feeling powerless to oppose their elders and advocate for their time to focus on their studies: “it does happen in lots of families, the issue of favouritism… you can do chores at home and they will always ask you to do the chores [again] even if you had done it… If you said you have done it, they will say you are rude because you are answering (back)” (Newcastle, 13–17 years).

At the macrosystem level were also narratives of gender based violence, domestic abuse, sexual violence, and a lack of personal safety for young women. Respondents explained that interlinked with interpersonal level stressors such as lack of psychosocial support and sparse supportive structures to support victims of violence, experiencing sexual violence results in heightened feelings of isolation and the sense of being unable to cope: “Many girls think that they’re alone and they face many bad things alone… Let’s just say as a girl you were raped… it will cause a very, very, very big stress for you… you are going to think a lot, you are going to think maybe you do not belong in this world, you are not part of this world… struggling is when a person can’t do anything, like that person is trying but is failing… that is what I mean about struggling… That’s when you realize that you need support… when tragedy happens…you will realize that you need supportive people around you” (Moretele, 13–17 years).

### Coping strategies and resilience enablers at various socio-ecological levels

#### Individual/intrapersonal resilience enablers.

Respondents described strategies they used as individuals to alleviate and cope with stressors including taking pleasure in the good things in life, such as good food, as a way of enjoying life and staying happy. In addition, making an effort to maintain a positive outlook and one’s own emotional stability was described as a way of ensuring a calm and level outlook on life. Compartmentalising parts of one’s life was also a strategy employed – making an effort to not let challenges from the home environment affect the school environment: “I am always a happy person; I am not a moody person. I do not bring my home issues here at school” (Newcastle, 13–17 years). A strategy respondents shared for maintaining a positive mindset included thinking positive thoughts and focusing on good things: “I am always a happy person, even my friends know that… I think happiness depends on your mind set, if you think about bad things then everything will turn out badly… It is important for a person to have a positive mind set, then you will likely to be happy. I think that helps because I always think about positive things” (Newcastle, 13–17 years).

Respondents described mental health self-care practices that they engaged in to cope with negative emotions, such as listening to music: “(The thing that makes me happy) is music, there is this one song that I like… even if there is something that hurts me, once I sing this song… That song relieves all the pains I had” (Newcastle, 13–17 years). Finding meaning in and relating to song lyrics was described as a way of finding comfort, self-awareness and understanding one’s own feelings: “I play music and write everything down… There is a song about ‘Depression’” (Newcastle, 13-17 years). In addition to listening to music, other recreational activities such as playing sports were also a strategy employed by some AGYW. Being physically active and getting sufficient sleep were also described as important for mental health: “(In order to face challenges)... I sleep after studying. I also listen to music to de-stress and go the field to play basketball” (Newcastle, 13-17 years). Additionally, the practice of writing down one’s feelings or journaling was described as a way to vent your emotions, without risking friends’ reactions or gossip: “I used to write down because it is not easy to share with your friends because they might share your story with other people” (Newcastle, 13–17 years).

### Microsystem/ interpersonal resilience enablers

At the interpersonal level, one key strategy related to seeking psychosocial and emotional support from others and talking about one’s feelings. These AGYW had trusting peers, family and broader community support with whom they could share with and seek guidance and support: “I do talk to my close friend… I talk to her about everything I need to” (Moretele, 13–17 years). In addition to seeking support from peers, respondents suggested that spending time with family and household members, and the feeling of having family support and a caring support network were powerful emotional well-being enablers: “(It makes me happy) to stay at home with my grandmother. And spending time on the phone (with friends), makes me happy” (Newcastle, 18 + years).

### Macrosystem/contextual resilience enablers

In the broader community setting extending beyond the family, respondents also spoke about religious/faith groups and gatherings as a way of mitigating stressors and negative emotions: “It helps me to go to church... I get peace at church” (Newcastle, 13–17 years). Social workers from the government’s Department of Social Development were described as an important support mechanism: “If I need to talk to social worker… I can call them and they are able to come and talk to me” (Moretele, 18years+). It was suggested that social workers provide important psychosocial support to AGYW, with those who are identified as being in need of additional help being referred to a mental health professional: “There are social workers… if you go to them and explain them to them your problems… There are those (girls) that are able… to talk… about their problems… so they get help… for others they are being provided with therapist” (Moretele, 13–17 years). However accessing public sector social workers is not always easy: “many girls in this community wanted to have access to social workers, but sometimes you can go to the social workers and you find that they are not there, they say ‘today they are not working’… let’s just say I borrowed money and said this weekend I’m going to the social workers, I’m going to explain to them my problem, but when I go there I do not find them… I find them that they are off (work) or maybe they moved them to another place and I don’t have money anymore” (Moretele, 13–17 years). In additional to public sector social workers, respondents explained that community-based organisations provide support to AGYW: “they (community-based organisations) also go around the families to children whom maybe they are not okay… there’s a social worker who can talk to them” (Moretele, 13–17 years).

### AGYW perceptions of the mental health impacts of intervention participation

In the intervention phase interviews, AGYW who had enrolled into the Imagine programme and had received services described various ways in which their participation in the programme components had positively impacted their mental health. Mentorship and support provided by Imagine Programme staff helped to build self-esteem: “they gave me courage, ‘yes you can do this’… they even motivated me… to speak out loud and stop being silent” (Moretele, 13–17 years). The interactions that intervention beneficiaries had with programme staff such as coaches, were described as a mechanism for sharing emotions and therefore freeing oneself of stress: “I was able to open up and share my story with my coach and that made me happy… I am a happy person, I’m free and I am enjoying the life I am living now” (Newcastle, 13–17 years). Support received from social workers employed by the Imagine Programme also helped those AGYW who had been struggling with mental health challenges and stress: “I went to see a social worker so that I can get encouragement, sometimes there is a time where you feel like you can give up” (Newcastle, 13–17 years).

The perception of having access to Imagine Programme social workers to speak to about challenges made AGYW happier and reduced their stress levels: “(What helped me)… it was an Imagine social worker… it is easier now that there are people I can talk to if I encounter any challenges” (Newcastle, 13–17 years). Respondents explained that since participating in the intervention, their perception of having access to psychosocial support had changed. Being able to access support from Imagine social workers and coaches meant that AGYW now felt less emotionally isolated*:* “Many of us girls have received assistance from this programme… Other girls like me did not have people they can talk to about the challenges they are facing… So since there is Imagine, others were able to talk to the social worker and the coaches” (Newcastle, 13–17 years*).*

One effect of participating in the programme described by AGYW was the feeling of knowing where to go for support and for reliable advice, which helped to reduce levels of stress and worry: “To be in that programme… it has helped a lot… I don’t even have worries because I know where I can look up to… people I interact with, those that I talk to… every time when I am not well I know where I should go, and they will give good advice” (Moretele, 13–17 years). In addition to the perception of receiving psychosocial support through the Imagine Programme, respondents described the sense of being able to talk about their feelings, which helped to lighten the burden of negative emotions: “When you take part in those activities you’re able to expose yourself, you become okay, you feel very alive… being around people and those activities made me feel light and very alive… It was good, after that (counselling) session I felt lighter, like I was able to move on… I vented out” (Moretele, 13–17 years).

In a powerful statement, one participant from Moretele articulated that prior to participating in the Imagine programme, she had never felt happy, as she had worried about her future and been stressed by her home environment. She reported that since participating in the Imagine Programme, she has felt happier than ever before: “(Participating in Imagine has affected my life) to be honest I have never been so happy in my life, just because I always worry or ask myself what is going to happen with me? Because of noise at home, the stress they give to me, sometimes I even think why am I still on earth… I don’t remember being happy. (But now) I’m happy” (Moretele, 13–17 years).

## Discussion

Drawing on Bronfenbrenner’s social-ecological model and Lazarus and Folkman’s theory of Stress and Coping as conceptual frameworks, our study describes the multi-level mental health stressors and resilience enablers from the perspective of adolescent girls and young women in South Africa.

Individual/ intrapersonal level stressors described by respondents included being critical of oneself, self-judgment, self-blame, low self-esteem, a lack of self-belief, lack of ability to articulate feelings, non-heteronormative sexuality and academic stress. Early childbearing, or being a young mother, was described as a key stressor in our data for several reasons: the self-perceived lack of skills and capacity to be a parent; feeling overwhelmed and unable to cope; challenges continuing education; and anxiety and stress caused by unexpected pregnancies. It is critical to consider the interconnectedness between mental health and sexual and reproductive health amongst South African AGYW, in what has been described as an SRH-mental health syndemic and ensure that interventions are responsive to these intersections [1].

Lacking hope for the future was also cited as a mental health stressor, particularly amongst AGYW who experience early pregnancy or childbearing. It was also suggested that loss of hope leads to destructive behaviours such as problematic alcohol use. Considering that low self-esteem and poor emotional regulation skills are associated with poor metal health amongst adolescent girls, and that agency, hope, self-esteem and self-efficacy, all of which are critical elements of an adolescent’s resilience and ability enact coping mechanisms, it is clear that interventions need to strengthen agency, emotional regulation and AGYW’s ability to draw on resources to respond to stressors [3,17,22,26]. Our findings suggest that mentorship and support provided through interventions such as the Imagine Programme can be successful in building AGYW self-esteem and fostering hope.

Intersecting at the individual and relational levels are the feelings of isolation and lack of psychosocial support experienced by AGYW, combined with the lack of the skills to communicate or articulate one’s mental health needs. The result of being unable to communicate feelings means that AGYW are emotionally isolated, unable to access support, which can lead to depression and suicide ideation. Respondents in our study described how access to coaches and social workers in the Imagine Programme increased their ability to access psychosocial support and build emotional regulation skills, thereby reducing feelings of emotional isolation, enhancing resilience and reducing negative mental health outcomes. These findings suggest that psychosocial components of combination interventions such as the Imagine Programme have the potential to positively impact AGYW mental health through increasing AGYW’s access to psychosocial support, building AGYW’s self-esteem and skills for articulating personal challenges and seeking support, and fostering hope, which in turn can positively impact AGYW’s capacity to cope with stressors, enhance resilience, and improve mental health and well-being. Given the importance of perceived social support to adolescents’ capacity for coping with stressors, interventions should include components to build adolescents’ self-efficacy, and teach skills and strategies for coping through learning to seek social support [27–29].

One salient stressor described by respondents related to academic stress, with AGYW describing how psychological distress was enhanced by their fear of failure and pressure to succeed, while at the same time lacking academic support. Anxiety and stress related to academic achievement combined with a lack of connectedness with and inability or unwillingness to seek support from teachers in AGYW narratives. Evidence suggests that school based-programmes can be effective in reducing adolescents’ exposure to academic stressors, and that teachers are well placed to support adolescents in coping with academic stress, but also to offer broader mental health support [4,30,31]. However, research from South Africa shows that whilst teachers are in a position to provide critical psychosocial and emotional support to adolescents and young people, there is a pervasive student-teacher disconnect characterised by a lack of effective communication and emotional support from teachers which negatively impacts young people’s mental health and academic achievement [31].

Relationships and dynamics within the peer group setting were also a key stressor described by respondents in our study. Peer pressure, and the desire to fit in and belong were a cause of stress and social anxiety. In the period of adolescence, social stressors caused by increasingly salient peer relationships and romantic relationships increase in importance, particularly for female adolescents [14,18,32]. The pressure to attain and maintain social standing and popularity within peer networks can cause AGYW psychological distress, and lead to increased risk behaviours [33]. Evidence shows that the association between stress in interpersonal contexts and negative mental health outcomes such as depression, are consistently higher for adolescent girls and young women as compared to their male counterparts; this gender difference is attributed to the higher value that adolescent girls place on their interpersonal relationships [34].

Concurrent with literature using the socioecological framing to examine adolescent mental health listing stressors in the home environment, such as emotional/physical abuse or neglect, and the absence of caring adults, respondents in our study described mental health stressors within the family, home, and household levels [3,13,22]. In particular, it was suggested that AGYW who experience parental neglect or abuse, and parents’/caregivers’ alcoholism face high levels of mental health stressors. In addition, situations of violence in the home, between parents, or having a parent as a victim of violence enhanced mental health stressors experienced by AGYW. Adverse childhood experiences, including personal experiences of gender-based violence and exposure to household dysfunction and abuse, have been robustly linked to a range of mental health challenges in adolescence, contributing to heightened vulnerability among AGYW [37,38]. In light of evidence suggesting that family context, particularly parental neglect or rejection, is one of the most salient factors in negative mental health stressors for adolescents and is consistently linked to psychological distress, mental health interventions for AGYW could focus on fostering positive interpersonal relationships and strengthening both formal and informal family and community level support and resilience enabling resources available to AGYW [4,6,27,32,39]. Considering that interpersonal mental health stressors for adolescents can include conflict at the family level, social isolation, peer rejection, and experiences of abuse, and that positive interpersonal relationships are drivers of mental well-being amongst adolescents, it is evident that interventions should include activities designed to facilitate social connection through mentoring, peer support groups and provide accessible psychosocial support [4,13,28,40].

Intersecting across individual, interpersonal and macro levels is the social context of heteronormativity – the privileging of heterosexuality as the normative, expected, acceptable sexuality – in many South African communities. This was apparent in respondents’ narratives of social discrimination relating to non-heterosexual sexualities being a mental health stressor, particularly in contexts of family rejection and community homophobia/homoprejudice. There has been limited research examining societal heteronormativity in South African society as a psychological stressor amongst adolescents who may not fit the heteronormative cisnormative expectations of communities and families, warranting further research into this area [41].

Our findings also suggest that gender inequities at the macro system level act as mental health stressors for AGYW. The burden of unpaid care work in households often falls on AGYW, who play key roles in caring for younger children, sick or elderly relatives and undertaking domestic labour in the household, often at the expense of their education and mental health. Research is limited on the way in which the gendered nature of domestic roles and the burden of care falling on AGYW to carry out unpaid household duties impacts on their mental health, particularly in low resourced settings, however evidence suggests a clear link between unpaid work and poor mental health amongst women [35]. As illustrated by our findings, it is likely that gender inequities and the unequal treatment of female children in households negatively impacts the mental health of AGYW, as has been described in resource poor settings in other parts of the world [36]. In order to bolster resilience and counteract the burden of care, systemic interventions should prioritise redistribution and recognition of reproductive labour, which could be achieved through expanding social protection mechanisms, such as access to social grants, food security and livelihood programmes, which may help to alleviate pressure on girls and young women [35].

Also at the macrosystem level lies the intersection between poverty and psychological distress in adolescents [5,22]. Respondents in our study articulated that circumstances of poverty and food insecurity serve as stressors to AGYW mental health. The association between household food insecurity and poor mental health outcomes among AGYW during the COVID-19 pandemic has been well-documented, highlighting the significant psychological burden food insecurity imposes on this vulnerable group [42]. Socio-economic adversity may also often overlap and intersect with other emotional and interpersonal stressors, particularly if there is abuse or violence in the household environment [5].

Socio-ecological resilience refers to the capacity of an individual to navigate and draw upon the psychological, social, cultural, and physical ‘resilience-enabling’ resources that can help to sustain their well-being in a context of exposure to significant adversity [38,43]. Our findings concur with evidence showing that resilience-enabling resources that adolescents draw upon most often are in the personal and relational domains [16]. Resilience resources at the individual level include self-esteem, positive thinking, and support-seeking, resilience resources at the interpersonal level include psychosocial support from family and community [28]. Adolescents with a strong sense of self have been shown to be more resilient [26]. Respondents in our study articulated that a sense of self-awareness and understanding facilitated coping with stressors, and described engaging in activities that enabled resilience such as making an effort to maintain a positive outlook, self-care, self-reflection and actively seeking support from others. It was not clear in our data why some individuals showed more resilience than others, however it is apparent that while some adolescents who live in multi-stressor environments manage to cope with stressors, others are less able to. In the evolution of resilience research, there is increasing recognition that instead of emotional resilience being an individual personality trait that is static, rather resilience appears to be a much more interactive and dynamic process [12,41,44].

Resilience enablers at the macrosystem level described in our study included supportive faith groups, communities and community-based organisations. Access to government social workers was a resilience enabler but was not always easy to obtain. Other macrosystem level resilience enablers could include social protection, food security and livelihood programmes, gender norm transformation and community engagement and GBV-sensitive crisis response; these would require robust legal and policy frameworks, alongside GBV-sensitive crisis response mechanisms that provide safe spaces tailored to AGYW, youth-centred emergency support, and accessible legal aid and psychosocial support for survivors [45]. For meaningful and effective gender transformations to take place, interventions need to be multi-level, reaching across individual, peer, family, and community spheres of influence [46].

Our findings can help to build an understanding of how and why some young people manage to employ coping strategies and psychological strengths to mitigate the negative impacts of stressors on their mental health and well-being, and identify factors and resources across various socio-ecological levels that bolster or hinder adolescents’ coping strategies and capacity for resilience, which is critical in order to support and enable them [3,5,16,47]. Respondents’ narratives about the impact of participating in the Imagine Programme build on evidence suggesting that interventions to support adolescents’ psychological well-being can help young people to develop a sense of agency and acquire strategies for regulating their emotions and coping with psychological distress through self-introspection and maintaining a sense of future purpose [26,48,49].

### Limitations

A limitation of this study that should be noted is that the sample of AGYW interviewed after the intervention implementation activities commenced was small, due to early closure of the study as a result of funding termination. Consequently, the ability to draw conclusions around the impact of the intervention on mental health of AGYW is limited. However, despite the small sample, the experiences and perceptions of these AGYW is valuable. Secondly, there may have been some scope for social desirability bias in reporting on the positive impact of the intervention, particularly if the respondents assumed some connection between the interviewers and the implementers, even though this was clearly stated in the consenting process.

## Conclusions

Adolescent girls and youth women in South Africa experience a range of intersecting and multi-level mental health stressors [22]. Although factors at the interpersonal level were described by respondents in our study as one of the key domains of negative mental health stressors, some AGYW also manage to draw on interpersonal resources to increase resilience and promote coping. Our findings concur with prior evidence suggesting that for young people in South Africa, interpersonal relationships are a key factor determining adolescents’ ability to cope with stressors, demonstrating that it is not only negative qualities in interpersonal relationships that are predictive of mental health outcomes, but also positive aspects that need to be capitalised upon [32]. The identification and recognition of mental health stressors in adolescents is the essential starting point, but delineating the strategies and interventions for coping with these stressors and supporting adolescents is a critical need. Individual level interventions need to be co-occurring alongside efforts to address stressors at other socioecological levels [3]. Moreover, our findings emphasise the importance of understanding how these individual and interpersonal level stressors are embedded within wider macrosystem structural contexts. AGYW’s interpersonal relationships are inextricably embedded within systemic stressors such as household poverty, food insecurity, gender inequities, and gender-based violence, which require systemic change, policy engagement and socio-cultural transformation to enable resilience. Mental health interventions for AGYW need to take a multi-level approach to enabling individual internal resilience by strengthening interpersonal, social, contextual and structural resilience resources available [38,42]. The findings of this study shed light on which stressor domains and resilience enabling resources are salient to the mental health and well-being of AGYW in these two South African communities, building a critical evidence base upon which to respond in a contextually appropriate way.
